# BDNF Haploinsufficiency Limits Voluntary Exercise-Induced Adult Hippocampal Neurogenesis

**DOI:** 10.3390/biology15171536

**Published:** 2026-09-04

**Authors:** Monique Klausch, Jens Schepers, Viola von Bohlen und Halbach, Oliver von Bohlen und Halbach

**Affiliations:** Institute of Anatomy and Cell Biology, University Medicine Greifswald, Friedrich-Loeffler Str. 23c, 17487 Greifswald, Germany; monique.klausch@uni-greifswald.de (M.K.); jens.schepers@online.de (J.S.); viola.bohlenundhalbach@uni-greifswald.de (V.v.B.u.H.)

**Keywords:** BDNF, doublecortin, neuronal plasticity, neurogenesis, running wheel, voluntary exercise

## Abstract

Adult hippocampal neurogenesis is involved in hippocampal plasticity and can be enhanced by voluntary physical activity, which also elevates “brain-derived neurotrophic factor” (BDNF) levels and improves memory performance. BDNF might either be essential for the maintenance of neurogenesis, or be necessary for mediating activity-dependent increases in neurogenesis. In order to get more insight on this, we examined male heterozygous BDNF-deficient (BDNF +/−) mice with or without access to a running wheel. Only males were analyzed, since sex steroids can interact with BDNF signaling pathways. Although male BDNF +/− mice exhibited a higher body weight as compared to age-matched controls (BDNF +/+), they did not differ in distance traveled, running time or running speed. We then analyzed whether changes in adult hippocampal neurogenesis can be observed. Under housing conditions without running wheels, the rates of basal adult hippocampal neurogenesis were not altered between genotypes. Interestingly, voluntary wheel running significantly increased neurogenesis in both groups; however, the magnitude was attenuated in BDNF +/− mice. These results indicate that, in adult male mice, reduced BDNF availability does not impair basal adult hippocampal neurogenesis, but limits the plasticity-related enhancement of neurogenesis induced by physical activity.

## Simple Summary

Regular exercise boosts the brain’s ability to generate new nerve cells in the postnatal hippocampus, a region which is important for learning and memory. Here, we analyzed whether a protein, called brain-derived neurotrophic factor, is needed for this effect. We studied six-month-old male mice with only one working copy of the *brain-derived neurotrophic factor gene* and compared them to normal mice, with or without access to a running wheel. Mice with the less brain-derived neurotrophic factor weighed a bit more and ran slightly differently. Without exercise, both groups showed similar numbers of new hippocampal cells. When given a running wheel, both groups increased the production of new neurons, but the increase was smaller in the mice with the reduced brain-derived neurotrophic factor. This suggests normal brain-derived neurotrophic factor levels are not required for maintaining baseline cell production, but brain-derived neurotrophic factor is important for the additional increase that comes from physical activity.

## 1. Introduction

Brain-derived neurotrophic factor (BDNF), as well as nerve-growth factor (NGF), neurotrophin-3 (NT-3) and neurotrophin 4/5 (NT4/5) are members of the neurotrophin family. These neurotrophins can bind and signal through different receptors that belong to the family of tropomyosin receptor kinases (trks). TrkA preferentially binds NGF, whereas BDNF and NT-4 bind to trkB and NT-3 mainly signals through trkC. In addition, all these neurotrophins can signal through a low affinity receptor, termed p75 neurotrophin receptor, shortly p75^NTR^ (see for details [1,2,3]). BDNF is expressed during embryonic development. Postnatally, the expression of BDNF persists in the brain and several other tissues. BDNF is thought to play a crucial role in neuronal and synaptic processes that are involved in mechanisms of learning and memory [4]. Thus, it is not surprising that polymorphisms in the human BDNF gene are associated with several neuropsychiatric disorders (such as major depression, schizophrenia or bipolar disorders) and neurodegenerative diseases (such as Parkinson’s or Alzheimer’s disease (see for details [1,5,6])).

A brain structure that is critically involved in different types of neuronal plasticity as well as in learning and memory is the hippocampus. Morphological correlates of neuronal plasticity can be seen in the hippocampus (on the level of dendritic spines [7]) or in the postnatal generation of new and functional neurons within the dentate gyrus (DG), termed adult hippocampal neurogenesis [8]. It is known that the rate of adult hippocampal neurogenesis can be increased by voluntary activity, for instance in rodents by voluntary wheel running. This does not only ameliorate adult hippocampal neurogenesis, but also improves memory functions and increases BDNF levels, at least in mice [9]. Thus, there seems to be a correlation between neuronal plasticity, learning and memory functions and BDNF levels. Indeed, reduced levels of BDNF have been associated with declines in memory functions also in humans (see, e.g., [10]). The Val66Met polymorphism in the BDNF gene affects BDNF secretion, hippocampal function and memory [11,12]. Moreover, polymorphisms in the BDNF gene have also been associated with poorer working memory and altered morphology of the hippocampus, cerebellum and frontal cortex in a Swedish elderly population [13]. Furthermore, there is evidence suggesting that this BDNF polymorphism may be involved even in learning disorders in some children [14]. This indicates that a correlation between neuronal plasticity, learning, and memory functions and BDNF levels exists.

Given the important role of BDNF on neuronal plasticity and the beneficial effects of voluntary wheel running on adult hippocampal neurogenesis, we analyzed heterozygous BDNF-deficient (BDNF +/−) mice in comparison to their wildtype littermates (BDNF +/+). In this study, we focused on adult male mice, since sex steroids can directly interact with BDNF signaling pathways, which may explain sex differences in neuroprotection, plasticity, and stress responses [15]. In addition, it has recently been shown that activity-dependent BDNF signaling pathways show sex-specific effects on the intrinsic excitability of pyramidal prefrontal neurons [16]. This suggests functional differences in synaptic plasticity, which may have an impact on neuronal plasticity and adult hippocampal neurogenesis. Therefore, we analyzed the basal rate of adult hippocampal neurogenesis in male heterozygous BDNF-deficient mice. Since running is a strong activator of hippocampal neurogenesis and learning capacities [17], we also analyzed the running induced effects on adult hippocampal neurogenesis.

## 2. Materials and Methods

Six-month-old male BDNF +/− mice and their age-matched control littermates (BDNF +/+) were used in this study. The mice were initially generated by Korte and coworkers in 1995 [18]. Mice were housed and bred at the “Zentrale Service- und Forschungseinrichtung für Versuchstiere” (ZSFV) of the University Medicine Greifswald, Germany. Voluntary running wheel experiments were performed at the animal facility of the Institute of Anatomy and Cell Biology of the University Medicine Greifswald, Germany. Since male mice were used in this study, the animals were kept individually. Animals were kept in a 12 h day–night cycle with food and water access ad libitum. All experiments were conducted in accordance with the legal requirements of the “Landesamt für Landwirtschaft, Lebensmittelsicherheit und Fischerei Mecklenburg-Vorpommerns” (LALLF-MV; approved laboratory animal application LALLF-MV 7221.3-1-016/22).

### 2.1. Determination of Body Weight and Feed Intake

Body weight of the mice was determined once per week with a precision scale (KS 36, Beurer, Ulm, Germany). Feed intake was determined by taring a beaker on the scale and adding food. The weekly feed intake was calculated as the difference between the amount supplied the previous week and the amount present in the trough at the time of measurement.

### 2.2. Running Wheel Experiments

The male mice were randomly assigned to either a baseline (“non-runner”, n = 15 per genotype) or running (“runner”, n = 10 per genotype) condition. Mice under the running condition had unlimited access to a running wheel (STARR Life Sciences Corp., Oakmont, PA, USA) for three weeks. Running distances and times were continuously recorded as revolutions of the wheel per 15 s measurement interval using a computerized tracking software VitalView Activity 1.1.14 (STARR Life Sciences Corp., Oakmont, PA, USA). The following parameters were analyzed:

- The distance covered (in km) was calculated for each day by multiplying the total number of daily wheel revolutions (U) by 34.87 cm (circumference of the wheel). The daily running time, expressed in hours (h), was calculated by multiplying the total number of daily active units by 15 s. An active unit corresponds to a 15 s measurement interval with ≥1 wheel revolution (U). Due to the running wheel installation being late in the morning, day 1 was excluded from statistical analysis.
- Each day was divided into a light and a dark phase according to the day–night rhythm. The running time, distance, and speed (in meters per minute (m/min)) were determined for each light/dark phase. Running speed was calculated using the mode, as it reflects the animal’s typical running speed more accurately compared to the mean or median [19].
- Since the mice were mainly active during the dark phase, each dark phase was divided into four equal quarters (Q1–Q4) and analyzed in more detail. The distance run per quarter relative to the total distance run during that dark phase was expressed as the percentage of activity (%) per quarter. The same methodological approach was used to calculate the proportional running time (%) per quarter. Furthermore, the running speed (m/min) was assessed for each quarter.

### 2.3. Determination of Adult Hippocampal Neurogenesis

Adult hippocampal neurogenesis was analyzed using the marker doublecortin (DCX). Neurogenesis was analyzed in non-runners (n = 10 per genotype) and runners (n = 5 per genotype). Animals were euthanized and then transcardially perfused, first with phosphate-buffered saline (PBS), followed by fixation with 4% paraformaldehyde (PFA). Brains were explanted and post-fixed in 4% PFA for at least 2 weeks. A total of 30 µm thick serial coronal brain sections were cut using a vibratome (VT 1000S, Leica, Wetzlar, Germany). The obtained sections were transferred into the wells of a 24-well plate (Falcon^®^, Corning Inc., Corning, NY, USA) filled with 20% ethanol. Next, the sections were mounted onto Superfrost slides (R. Langenbrinck GmbH, Emmendingen, Germany), air-dried and stained with an antibody directed against DCX. For details see [20]. In brief: after antigen retrieval, sections were incubated in a solution (3% normal goat serum (NGS) + 0.1% Triton X-100 in PBS) containing the primary monoclonal anti-DCX antibody (Santa Cruz Biotechnology, Heidelberg, Germany; sc-271390; 1:100) overnight at 4 °C. After rinsing in PBS, sections were incubated in a solution composed of 3% NGS, 0.1% Triton X-100, and biotinylated goat IgG (Jackson ImmunoResearch, Ely, UK; 115-065-146; 1:200) in PBS for 2 h. After a further rinsing step, sections were incubated with Cy3-conjugated streptavidin (Jackson ImmunoResearch, UK; 016-160-084; 1:2000 in PBS) for 2 h. For visualization of cell nuclei, counterstaining with 4′,6-diamidine-2-phenylindole (DAPI (Sigma-Aldrich Chemie GmbH, Taufkirchen, Germany) diluted 1:10,000 in A. bidest.) was performed. Slides were covered with Mowiol (Mowiol 4-88, Carl Roth GmbH & Co. KG, Karlsruhe, Germany) and stored at 4 °C in the dark. Immunoreactive cells were counted in each third section throughout the total rostro–caudal axis of the DG. The total number of labeled cells was estimated by multiplying cell counts by three. No guard zones were used, since their use can bias even optical disector counting [21]. Images were captured using a BX63 microscope equipped with a DP-80 camera together with the software CellSens Dimensions 1.13 (all from Olympus, Hamburg, Germany). An image-stack (step width in the *z*-axis 0.3 µm) was generated and a Wiener-filter was used for deconvolution.

### 2.4. Statistical Analysis

For statistical analysis, GraphPad Prism version 5 for Windows (GraphPad Software, Boston, MA, USA, www.graphpad.com) was used. The level of significance was set to *p* < 0.05. First, it was evaluated whether the data followed a normal distribution. When this requirement was not met, non-parametric statistical tests were used. If the data were normally distributed, tests based on the assumption of normality were applied. Mann–Whitney U-test was applied when comparing two independent groups. As a non-parametric test for more than two groups, a Kruskal–Wallis test followed by Dunn’s multiple comparison test was used. To determine the main effects and their interactions, two-way ANOVA followed by Tukey’s multiple comparison test was used.

Data were expressed as mean ± standard error of the mean (SEM). Graphs were shown either as box and whisker blots, with the whiskers representing minimum and maximum values, or as bar charts (mean ± SEM).

## 3. Results

### 3.1. Body Weight and Feed Intake

**Figure 1:**
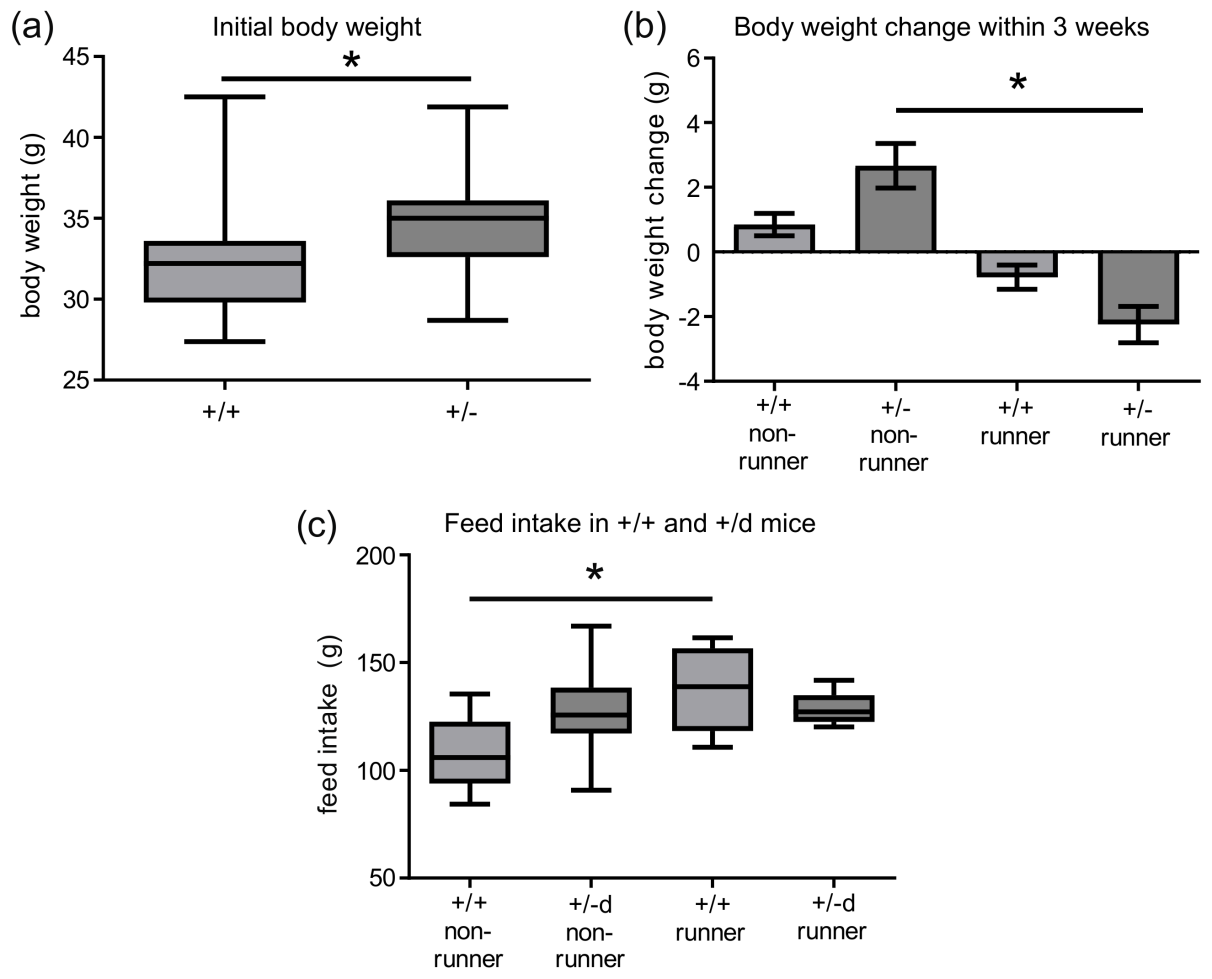
Body weight and feed intake. Initial body weight (g) of six-month-old male heterozygous BDNF-deficient mice (+/−) and their wildtype littermates (+/+) prior to running wheel experiments. The body weight of the six-month-old male +/+ and +/− mice was determined. The different groups of mice differ in the initial weight in the beginning of the experiment (**a**). Body weight change (g) in BDNF +/+ and BDNF +/− mice with unlimited access to a running wheel (runner; n = 10 per genotype) or without access (non-runner; n = 15 per genotype; two-way ANOVA; (**b**)). The total feed intake (g) during the three weeks of testing significantly differs in the +/+ mice, but not in heterozygous BDNF-deficient mice. The wildtype runners consume more food than their genotypically identical non-runners (runner; n = 10 per genotype; non-runner; n = 15 per genotype; two-way ANOVA; (**c**)). * *p* < 0.05.

Prior to the start of a running wheel experiment, the body weights of the heterozygous BDNF (BDNF +/−) and wildtype (BDNF +/+) mice were measured. The initial body weight significantly differed between the two genotypes. The mice exhibited a significantly increased body weight as compared to controls (+/+: 31.8 ± 0.6 g vs. +/−: 35.1 ± 0.75 g; *p* = 0.0003, Mann–Whitney Test; Figure 1a). Mice of each genotype were randomly assigned to either a runner group (access to a running wheel, termed “runners”, n = 10 per genotype) or non-runner group (no access to a running wheel, termed “non-runners”, n = 15 per genotype). After three weeks, runner mice of both genotypes lost weight and non-runners gained weight. BDNF +/+ runners showed a mild but not significant decrease in body weight as compared to BDNF +/+ non-runners. In contrast, in the group of BDNF +/− mice a significant difference was observed in weight loss of the runners compared to non-running mice (+/+ runner: −0.77 ± 0.23 g; +/+ non-runner: 0.79 ± 0.24 g; +/− runner: −2.0 ± 0.54 g; +/− non-runner: 2.23 ± 0.53 g; Figure 1b).

Feed intake was measured over the course of the running wheel experiments. Feed intake in the BDNF +/− mice of the non-runner group and runner group was not significantly altered. Conversely, BDNF +/+ runners consumed significantly larger amounts of food compared to BDNF +/+ non-runners. Interestingly, non-running heterozygous BDNF-deficient mice displayed a slightly, but insignificant higher feed intake compared to non-running control mice (+/+ non-runner: 105.1 ± 3.77 g; +/+ runner: 136.2 ± 5.76 g; +/− non-runner: 124.1 ± 4.82 g; +/− runner: 121.5 ± 3.41 g; Figure 1c).

### 3.2. Voluntary Wheel Running Behavior

**Figure 2:**
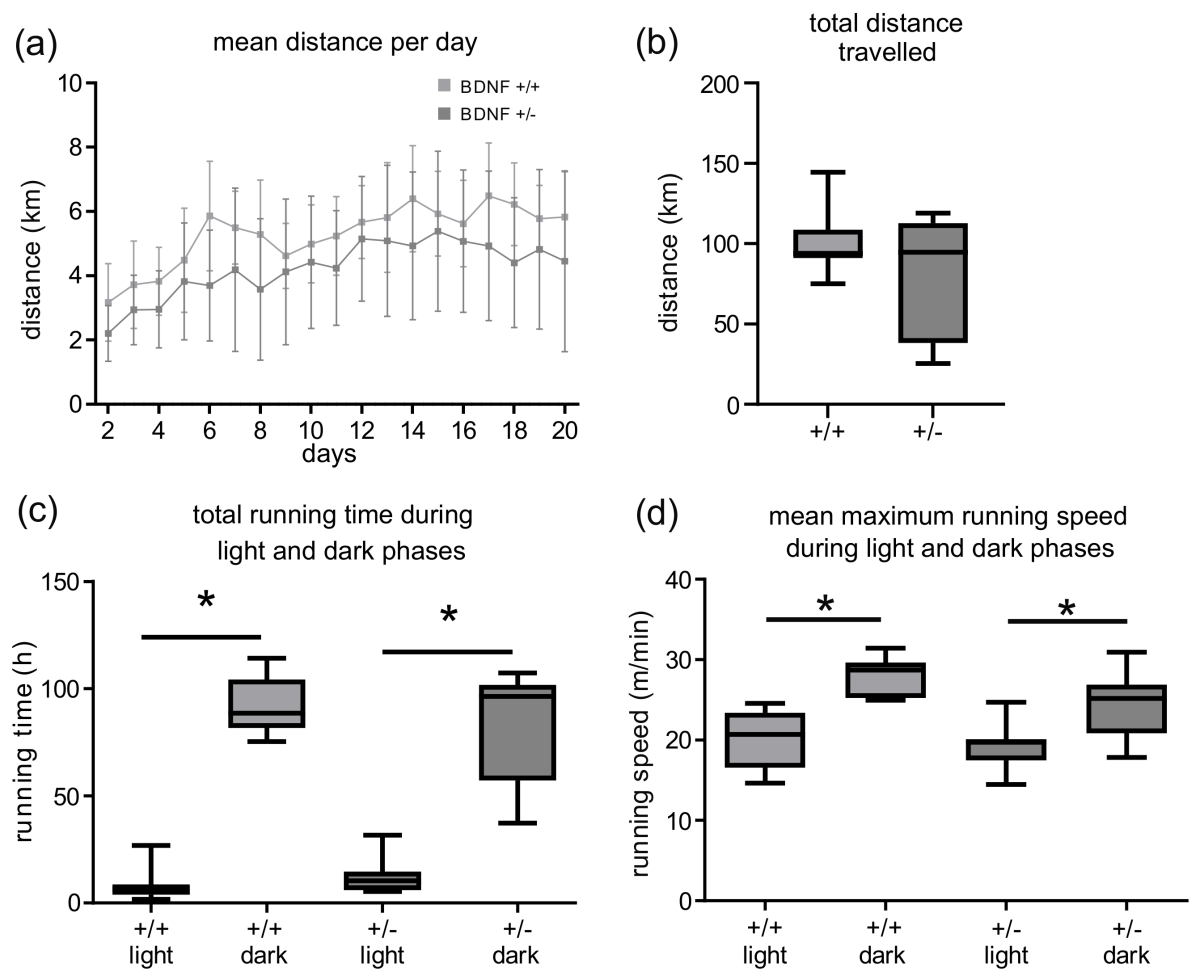
Running behavior during the light and dark phases. The distance (km) the mice ran in the running wheel was monitored during the test phase for “runners” of the BDNF +/+ and BDNF +/− mice (**a**). The mean total distance (in km) was comparable for both genotypes, with no major difference (Mann–Whitney *p* > 0.05), although a greater variability was seen in the group of the BDNF +/− mice (**b**). Both genotypes were more active during the dark phases than during the light phases. Thus, the running time was higher during the dark phases as compared to the light phases (**c**) as well as the maximum running speed (**d**). Concerning this behavior, no significant difference was observed between the genotypes (for (**c**,**d**), two-way ANOVAs followed by Tukey’s multiple comparison test were used for statistical analyses). * *p* < 0.05.

We monitored the voluntary wheel running behavior of the BDNF +/+ and BDNF +/− mice (n = 10 per genotype). At first glance, it appears as if the male BDNF +/− mice traveled less than the male BDNF +/+ mice (Figure 2a). Indeed, no significant difference in the total distance traveled (+/+: 103.0 ± 5.89 km; +/−: 85.89 ± 10.17 km; *p* = 0.579; Mann–Whitney U-test; Figure 2b) and in the total time the animals used the running wheel (+/+: 97.62 ± 5.70 h; +/−: 92.25 ± 8.99 h; *p* = 0.853; Mann–Whitney U-test) could be observed.

Furthermore, no differences in running behavior were observed between the two genotypes during the light and dark phases, and both genotypes exhibited their highest level of perceptual activity during the dark phase (+/+ light: 8.08 ± 2.24 h; +/+ dark: 92.84 ± 4.31 h; +/− light 11.82 ± 2.49 h; +/− dark: 82.47 ± 8.34 h; Figure 2c). Moreover, the absolute distances during the dark phases as well as the light phases also did not differ significantly between both genotypes. The mean speed did not differ significantly between the genotypes, but the BDNF +/+ mice are significantly (*p* = 0.001; two-way ANOVA followed by Tukey’s multiple comparison test) more active during the dark phases related to the light phases (+/+ light: 12.55 ± 1.258 m/min; +/+ dark: 19.92 ± 0.684 m/min; +/− light: 12.54 ± 0.837 m/min; +/− dark: 17.29 ± 1.044 m/min). Somewhat comparable to that, the maximum running speed in both genotypes was significantly higher in the dark phases than in the light phases, but without a significant difference between the genotypes (+/+ light: 20.66 ± 1.07 m/min; +/− light: 19.21 ± 0.83 m/min; +/+ dark: 28.03 ± 0.71 m/min; +/− dark: 24.23 ± 1.25 m/min; Figure 2d).

**Figure 3:**
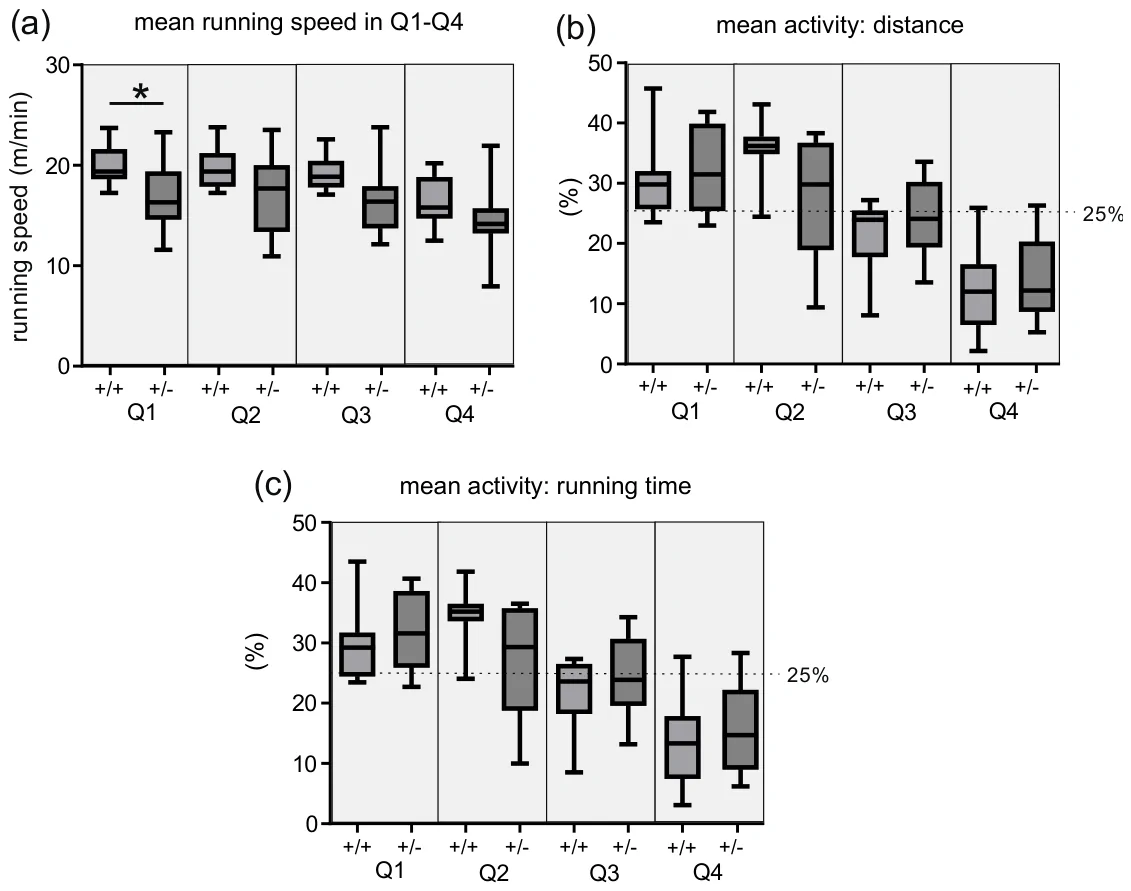
Activity patterns in the four quarters (Q1–Q4) of the dark phase. The mean running speed (m/min) is reduced in BDNF +/− mice as compared to BDNF +/+ mice, especially in the first quarter of the dark phase. However, exclusively in Q1 a significant difference between the genotypes could be observed (**a**). The mean activity, which was calculated as (**b**) distance traveled or (**c**) running time per quarter relative to the total distance traveled in the dark phase, was analyzed. The BDNF +/− mice did not differ significantly from the BDNF +/+ mice in the distance traveled within the different quarters, but a significant shorter distance was observed from the first (Q1) to the last quarter (Q4) (activity in %). In general, a reduction in the activity was observed from the first to the last quarter (**b**,**c**). For statistical analyses on the effects of the genotype in phase Q1–Q4, as shown in figures (**a**–**c**), Mann–Whitney U-tests were used. * *p* < 0.05.

Since the mice were mainly active during the dark phases, we analyzed the running behavior during that phase in more detail. We therefore subdivided the dark phase into four equal quarters and analyzed them separately for running speed (Figure 3a). Next, we determined the distance traveled during the quarters as an indicator of mean activity, which was calculated as distance traveled per quarter relative to the total distance traveled in the dark phase (Figure 3b). As a further indicator of mean activity, the mean running time was analyzed. This parameter was calculated as running time per quarter relative to the total running time in the dark phase (Figure 3c). These analyses showed that the mice were most active in the first quarters, which differed significantly from the last quarter. In the first quarter (Q1) BDNF +/− mice exhibited a significantly reduced running speed compared to BDNF +/+ mice (Figure 3a). In all other quarters (Q2–Q4), no significant differences were detected. The quarter-based analysis of running distance and running time during the dark phase revealed that both genotypes behaved comparably. Among others, both genotypes displayed a reduction in their activity from the first to the last quarters (Figure 3b,c).

### 3.3. Adult Hippocampal Neurogenesis

**Figure 4:**
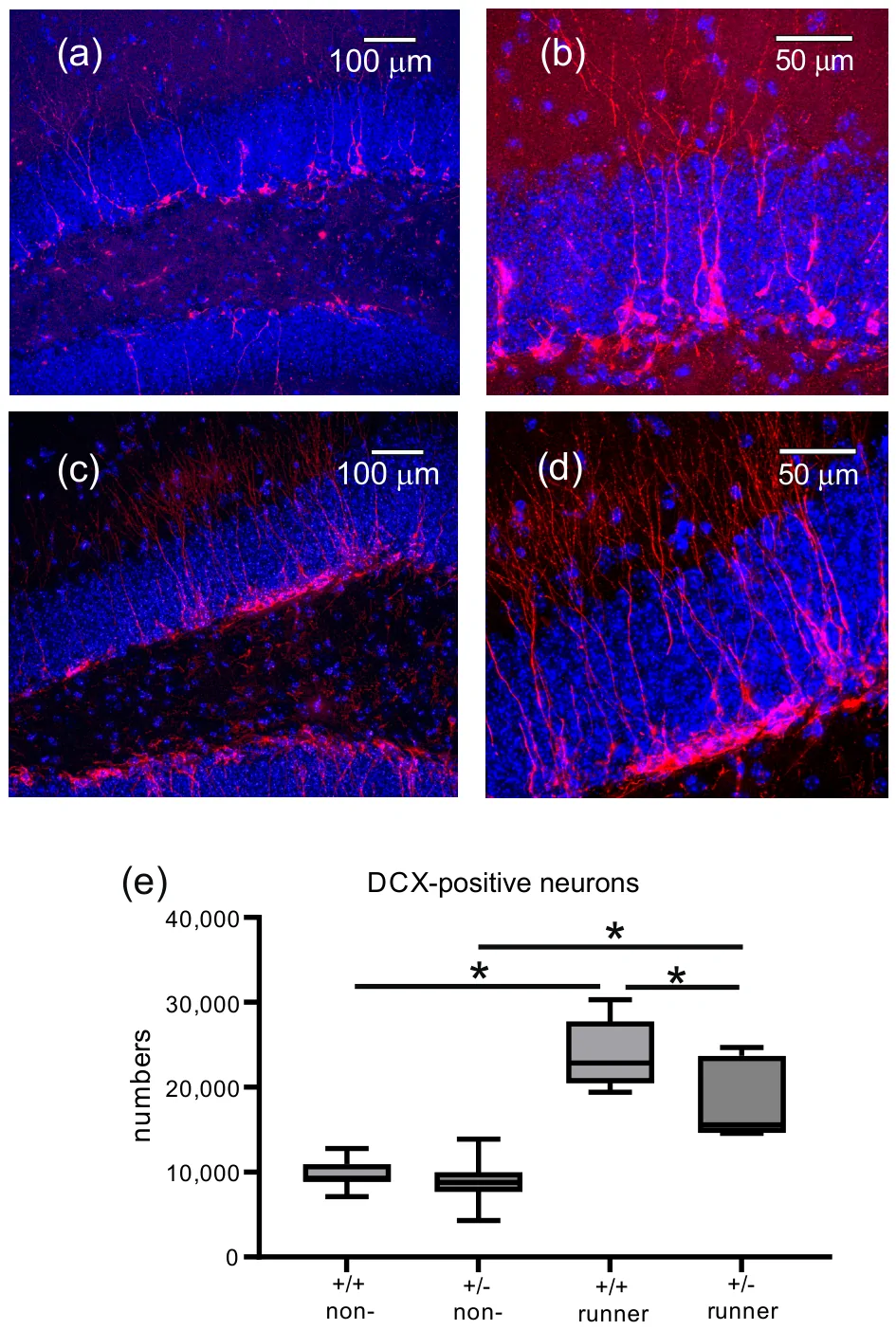
Analysis of the number of cells in the DG that expressed doublecortin (DCX). Example of a DCX-staining in the dentate gyrus (DG) of a non-runner mouse is shown in (**a**). DAPI (in blue) was used to visualize cell nuclei. DCX-immunoreactivity is shown in red. A higher magnification of an area seen in (**a**) shows that DCX-immunolabelling is not restricted to the cell body of newly formed neurons, but is also present in the dendritic processes that extend to the molecular layer (**b**). Example of a DCX-staining in the dentate gyrus of a runner mouse. DAPI (in blue) was used to visualize cell nuclei. DCX-immunoreactivity is shown in red (**c**). A higher magnification of an area seen in (**c**) is shown in (**d**). The number of DCX-positive cells is not different between BDNF +/− and BDNF +/+ non-runners (**e**). Running induces an increase in the number of doublecortin positive cells in both BDNF +/− and BDNF +/+ male mice; however, this running-induced increase was significantly higher in wildtype mice as compared to heterozygous BDNF-deficient mice (Kruskal–Wallis test followed by Dunn’s multiple comparison test was used for statistical analyses). * *p* < 0.05.

Since voluntary wheel running is a strong activator of adult hippocampal neurogenesis, we used DCX, a marker of newly generated young neurons, to monitor changes in adult hippocampal neurogenesis (Figure 4a–d).

Under baseline, i.e., non-running conditions, a low rate of ongoing adult hippocampal neurogenesis can be observed (Figure 4a,b). In contrast, voluntary wheel running for about three weeks induces a strong increase in the numbers of DCX-positive cells (Figure 4c,d). To obtain a better and more detailed insight into the plasticity-driven modulation of adult hippocampal neurogenesis, we estimated the numbers of DCX-positive neurons for non-runners (n = 10) and runners (n = 5) of both genotypes. Both BDNF +/+ and BDNF +/− mice showed comparable numbers of DCX-expressing neurons under baseline conditions (+/+ non-runner: 9686 ± 495 cells; +/− non-runner: 8897 ± 777 cells, Figure 4e). Consistent with the effects described in the literature, voluntary wheel running was found to increase the number of DCX-positive neurons in BDNF +/+ mice. Likewise, we also noted an increase in the rate of adult hippocampal neurogenesis in the BDNF +/− mice. However, the magnitude of this increase in the number of DCX-positive cells was significantly smaller in the six-month-old male BDNF +/− mice as compared to the BDNF +/+ mice (+/+ runner: 23,864 ± 1865 cells; +/− non-runner: 18,455 ± 2165 cells).

## 4. Discussion

In recent decades, several studies have focused on the role of BDNF on adult hippocampal neurogenesis under normal housing conditions and enriched environment (EE, for example by using larger cage containing differently shaped objects and running wheels) with varying outcomes. Recently, it has been shown that inhibition of BDNF/TrkB signaling via administration of the small molecule ANA-12 was sufficient to completely suppress hippocampal neurogenesis in a mouse model of EE [22]. In a study from 2008, BDNF-mutants of both sexes, averaging 8–12 weeks of age, in which BDNF stores in the brain had been depleted postnatally, were examined for adult hippocampal neurogenesis and compared to control littermates. These mutant mice showed an increase in the number of BrdU-positive cells (BrdU is a cell proliferation marker) of about 40% [23]. In 2002, Lee et al., investigated two months old male heterozygous BDNF-deficient mice, which exhibited a lower number of BrdU-positive cells in the DG in comparison to control mice. Based on that, they concluded that BDNF-signaling is required for the maintenance of basal levels of neuronal stem cell proliferation in the DG [24]. In addition, they could show that dietary restriction can counteract the adverse effect of reduced levels of BDNF on proliferation. In 2006, Rossi et al. analyzed four-month-old heterozygous BDNF-knockout mice and their control littermates and the effect of EE (i.e., a larger cage, containing several food hoppers, a running wheel and differently shaped objects) over a period of eight weeks. Under non-EE conditions, the wildtype and BDNF +/− mice displayed nearly the same basal levels of cell proliferation; however, survival of newly generated cells was significantly improved by EE in wildtype mice, but not in BDNF +/− mice [25]. In 2011, Jha et al. analyzed two-to-five-months-old male BDNF-KIV mice (these mice lack promoter IV driven BDNF-expression) and their controls in normal and EE conditions and found that the numbers of surviving progenitors were reduced in BDNF-KIV mice and that EE treatment increased cell proliferation in BDNF-KIV mice [26]. In summary, the experimental settings differ with respect to the mouse model as well as the age and sex of the animals used. These differences may account for the different results obtained. Concerning EE, it has been shown that cell proliferation, neuronal survival and neurotrophin levels were enhanced only when running wheels were available, suggesting that physical activity is the key factor mediating the increase in BDNF levels and adult hippocampal neurogenesis [27]. The positive effects of exercise may not be restricted to adult hippocampal neurogenesis but may also lead to beneficial changes in brain morphology, thereby mediating the positive effects on learning and memory [28].

Since BDNF is thought to play fundamental roles in neuronal and synaptic plasticity, we analyzed adult male BDNF +/− mice under baseline conditions as well as under conditions in which they had additional unlimited access to a running wheel. We previously reported an increase in the body weight of the BDNF +/− non-runner mice over time [29]. We found that male heterozygous BDNF-deficient mice exhibited a measurable increase in body weight over the three-week observation period. Interestingly, the +/+ runners showed only a small weight loss when they were allowed to use a running wheel, whereas this weight loss was significant in the BDNF +/− runner group. This could be an interesting side effect for future studies, since, in humans, an association between BDNF polymorphisms and obesity is frequently reported [30,31,32]. Thus, although BDNF +/+ and BDNF +/− runners did not differ in feed intake, BDNF +/− runners lost significantly more weight compared to their non-runners. This observation was not found in BDNF +/+ mice. These findings may indicate a differentially regulated energy homeostasis in male BDNF +/− mice compared control mice. Concerning the BDNF +/− mice, it recently has been shown that they not only develop signs of obesity, but also non-alcoholic steatohepatitis and the authors of that study suggest that BDNF might play a role in metabolic regulation through the control of appetite in the hypothalamus [33]. However, to analyze this in detail, mice with a conditional brain specific deletion of BDNF would be required.

It has been reported that the circadian rhythm affects BDNF levels in the brain and vice versa [34,35]. In addition, Phillips and coworkers demonstrated in 2022 that the Val66Met BDNF polymorphism modulates the effects of circadian desynchronization on activity and sleep in mice [36]. However, when comparing the running behavior of age-matched six months old male BDNF +/+ and BDNF +/− mice, we did not detect significant differences in their circadian rhythm. Surprisingly, the BDNF-deficiency and the higher initial body weight of the BDNF +/− mice did not seem to influence their general running behavior or their drive for voluntary exercise. We observed no significant differences in their distance traveled, running times or running speed during the total dark or light phase. Therefore, BDNF +/+ and BDNF +/− mice exhibited comparable levels of voluntary exercise, a finding that is particularly noteworthy in light of the results from the adult hippocampal neurogenesis experiments. Thus, a strong impact on adult hippocampal neurogenesis due to different running behavior of the two groups of mice can be ruled out. Under baseline conditions, six-month old male heterozygous BDNF-deficient mice displayed no significant difference in the number of DCX-positive neurons in the DG as compared to age-matched male controls. This hints that reduced levels of BDNF may not affect basal adult hippocampal neurogenesis. Interestingly, under similar levels of physical activity, we observed an increase in the number of DCX-positive cells in both genotypes, although this increase was less pronounced in BDNF +/- mice than in BDNF +/+ mice. However, it should be emphasized that DCX is a marker of immature neurons, and does not separately assess proliferation, differentiation and survival. For getting insight in these processes, other additional markers should be used [37].

This finding presented here suggests that reduced levels of BDNF are involved in plastic neurogenesis in the adult brain. This indicates that reduced BDNF levels have an impact on morphological and physiological changes related to neuronal and/or synaptic plasticity. Aside from adult hippocampal neurogenesis, changes in the shape and densities of dendritic spines are hallmarks of neuronal plasticity. BDNF is also known as a modulator of spine densities in the hippocampus [38] and BDNF is upregulated in the hippocampus during certain types of learning [39]. Long-term potentiation (LTP) is an electrophysiological model of activity-dependent synaptic plasticity that involves the persistent enhancement of excitatory neurotransmission. LTP can be induced in the hippocampus by electric stimulation, for example by theta burst stimulation [40]. LTP in the hippocampus is impaired in BDNF +/− mice [41], indicating that BDNF might have a role in synaptic plasticity. In addition, BDNF infusion was found to lead to a marked enhancement of hippocampal LTP [42]. These findings, together with the alterations in adult neurogenesis after voluntary wheel running seen in the adult male mice in this study, indicate that BDNF is a modulator of plasticity in the adult brain.

However, this might be different in female BDNF +/− mice, since sex steroids can interact directly with BDNF-signaling pathways [15]. Moreover, hippocampal dendritic spines, which are also critically involved in processes associated with learning and memory, differ in their morphology in a sex-dependent manner [43]. In this study, it was shown that heterozygous BDNF-deficiency has an impact upon the plastic modulation of adult hippocampal neurogenesis, at least in male mice. This could also explain why various studies investigating different BDNF-deficient mouse models without differentiating between the sexes, either observed differences or failed to detect any differences in adult hippocampal neurogenesis. Therefore, further sex-specific research is needed as sex but also age and different mouse models of BDNF-deficiency can lead to conflicting results on adult hippocampal neurogenesis, as previously stated.

## 5. Conclusions

Taken together, our findings indicate that in adult male mice, reduced BDNF availability does not impair basal baseline adult hippocampal neurogenesis, but limits the plasticity-related enhancement of neurogenesis induced by physical activity.

## Data Availability

The data presented in this study are available on request from the corresponding author.

## Abbreviations

The following abbreviations are used in this manuscript:

BDNF	Brain-derived neurotrophic factor
DCX	Doublecortin
DG	Dentate gyrus
EE	Enriched environment
NGF	Nerve-growth factor
NGS	Normal goat serum
NT	Neurotrophin
PBS	Phosphate-buffered saline
PFA	Paraformaldehyde
SEM	Standard error of the mean
Trk	Tropomyosin receptor kinase
U	Wheel revolution
Q	Quarter
